# Definition of Compartment Based Radical Surgery in Uterine Cancer—Part I: Therapeutic Pelvic and Periaortic Lymphadenectomy by Michael Höckel Translated to Robotic Surgery 

**DOI:** 10.1155/2013/297921

**Published:** 2013-03-25

**Authors:** Rainer Kimmig, Antonella Iannaccone, Paul Buderath, Bahriye Aktas, Pauline Wimberger, Martin Heubner

**Affiliations:** Department Gynecology and Obstetrics University Clinic Essen, West German Cancer Center, University Duisburg-Essen, Hufelandstraße 55, 45147 Essen, Germany

## Abstract

*Objective.* To define compartment based therapeutic pelvic and periaortic lymphadenectomy in cervical and endometrial cancer. Compartment based oncologic surgery appears to be favorable for patients in terms of radicality as well as complication rates, and the same appears to be true for robotic surgery. We describe a method of robotically assisted compartment based lymphadenectomy step by step in uterine cancer and demonstrate feasibility data from 35 patients. *Methods*. Patients with the diagnosis of endometrial (*n* = 16) or cervical (*n* = 19) cancer were included. Patients were treated by rTMMR (robotic total mesometrial resection) or rPMMR (robotic peritoneal mesometrial resection) and pelvic or pelvic/periaortic rtLNE (robotic therapeutic lymphadenectomy) with cervical cancer FIGO IB-IIA or endometrial cancer FIGO I-III. *Results*. No transition to open surgery was necessary. Complication rates were 13% for endometrial cancer and 21% for cervical cancer. Within follow-up time median (22/20) month we noted 1 recurrence of cervical cancer and 2 endometrial cancer recurrences. *Conclusions*. We conclude that compartment based rtLNE is a feasible and safe technique for the treatment of uterine cancers and is favorable in aspects of radicality and complication rates. It should be analyzed in multicenter studies with extended followup on the basis of the described technique.

## 1. Introduction

Lymphadenectomy in gynecological cancer is intensively discussed with respect to prognostic, predictive, and therapeutic aspects [1–5]. The prognostic and predictive approach aims primarily on detection of node metastases without inducing additional relevant morbidity. Thus, the sentinel node concept and different types of not well defined “staging lymphadenectomies” were developed. The therapeutic approach aims on removal of all nodes of certain lymph basins and the intercalated nodes at risk, implying cure in case of involvement. However, for potential surgical cure of a patient with lymph node involvement the nodes of risk have to be defined and removed completely. There is evidence that tumor spread is bound to permissive ontogenetic compartments for a long time [6]. This has first been demonstrated in rectal cancer [7] resulting in modification of surgical treatment by “Total Mesorectal Excision (TME)” [8] and later in cervical cancer resulting in “Total Mesometrial Resection (TMMR)” [9, 10]. The same tumor permissive or inhibitory compartment mechanisms related to embryologically derived compartments seem to be true for lymph basins as demonstrated by Höckel for lymphatic spread in cervical cancer. In his monocentric prospective study overall and five-year relapse free survival was 96% and 94%, respectively, for 305 patients stages Ib to IIb (71 node positive) following TMMR and therapeutic lymphadenectomy without any adjuvant irradiation [3]. It seems to be mandatory to remove the lymph node regions at risk completely, which can be defined by the pattern of lymph node metastasis and recurrent disease, respectively, in correlation to embryological development of anatomical regions. 

Robotic surgery enables us to develop and dissect structures with extremely increased accuracy, thus allowing to prepare and free the compartments completely of lymphatic tissue without injuring adjacent compartments by respecting the filmy septa at the compartment borders. Due to the excellent 3D/HD visualization surgical technique may be described step by step and may precisely be reproduced and documented in each patient. The authors translated Höckel's compartment based method of “therapeutic lymphadenectomy,” developed for open surgery of uterine cancer, to a robotically assisted approach, which will be described in the following step by step.

## 2. Methods

### 2.1. Surgical Technique

The first author learned the surgical technique of TMMR and tLNE participating in Leipzig School of Surgery in 2006. All radical hysterectomies were consequently performed using the nerve-sparing technique of TMMR and tLNE, if adequate. In 2010, robotically assisted laparoscopic surgery was implemented using a da Vinci Surgical System (Intuitive Surgical Inc., Sunnyvale, CA, USA). The principles of surgical steps were systematically translated to the robotic surgery and optimized to guarantee the same radicalness compared to open surgery but maintain the advantages of an endoscopic approach. The different steps were discussed with Höckel by video sequences. Finally, Höckel participated in the surgery in Essen and confirmed equality of the robotic approach with respect to his operation defined by open surgery. The resulting technique of rtLNE will be described in detail for the firsttime in international literature. The technique with respect to cervicalcancer has been already described in German only, but without reporting anyclinical data [11].

Patient preparation is identical to laparoscopy with deep Trendelenburg positioning. Trocars are positioned 23–25 cm above the symphysis (camera), two lateral robotic trocars about 5 cm–10 cm above the upper anterior iliac spine, one additional robotic trocar on the left between camera trocar and left lateral trocar, one assistant trocar of 10 mm diameter on the right side between camera trocar and right lateral trocar. The space in between the trocar incisions was at least 10 cm to ensure adequate mobility.

### 2.2. Patients and Specimens

As a first proof of feasibility, 35 patients were treated by rTMMR or rPMMR and rtLNE with cervical cancer FIGO IB-IIa or endometrial cancer FIGO I-III. Therapeutic lymphadenectomy was performed by “robotic surgery” in analogy to the procedure described by Höckel [3, 6]. Thus, the clearance of defined lymph basins and the intercalated nodes downstream to the Müllerian compartment were defined to be a superior quality parameter compared to the number of removed lymph nodes. These include for cervical cancer: mesometrial nodes (mm), paravisceral nodes (internal iliac nodes including gluteal and rectal nodes, pv), external iliac nodes (ei), common iliac nodes (ci), and presacral/subaortic nodes (ps) on both sides. In endometrial cancer, there will be no involvement of gluteal and rectal nodes but of periaortic nodes including inframesenteric (im) and supramesenteric/infrarenal (sm/ir) nodes, since primary lymph basins are reached via ovarian vessels directly. Perioperative morbidity and early postoperative morbidity were analyzed. We noted whether there was the need of perioperative blood transfusions and the length of hospitalization. In addition, the tumor related outcome was recorded.

### 2.3. Statistical Analysis

Analysis of clinical and histopathological data was performed using and SPSS17.0 for Macintosh (SPSS, Chicago, IL, USA). We conducted a descriptive analysis only, considering the limited number of patients and the explorative character of this study.

### 2.4. Technique and Results

**Figure 1:**
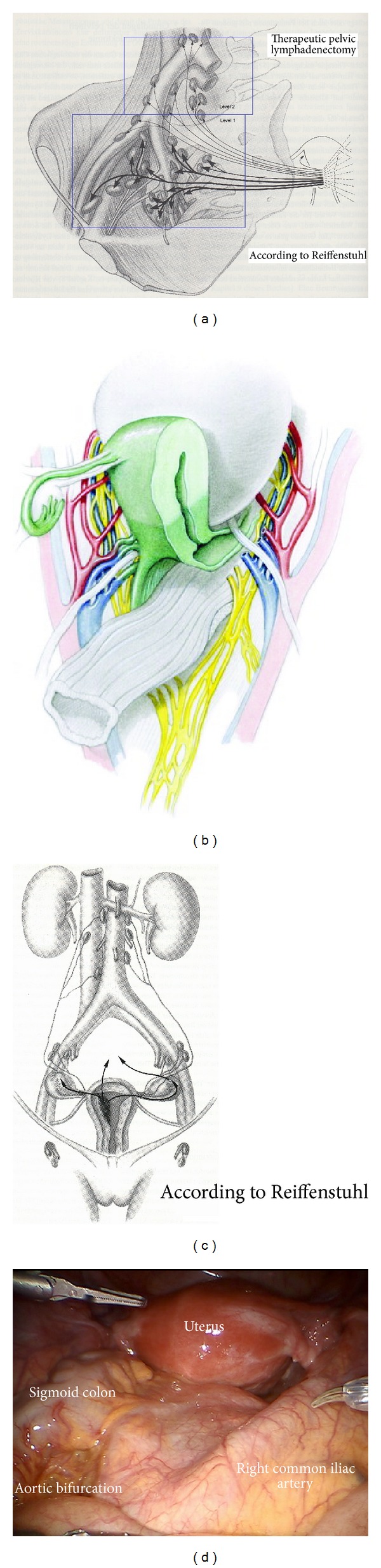
(a) Pelvic lymphatic basins of the uterus (from Hepp et al., Lymphonodektomie in der gynäkologischen Onkologie. Indikation, Technik und Konsequenzen für die Therapieplanung Urban and Schwarzenberg 1988 with permission from ELSEVIER GmbH, Urban and Fischer Verlag) [12]. (b) The structures of the female genital tract with reference to the embryologic (Muellerian) compartment (green) (from Höckel M. Do we need a new classification for radical hysterectomy? Insights in surgical anatomy and local tumor spread from human embryology. Gynecol Oncol. 2007 Oct; 107 (1 Suppl 1): 106–112 with permission from ELSEVIER) [13]. (c) Upper lymphatic basins of the uterine corpus and ovaries (from Hepp et al., Lymphonodektomie in der gynäkologischen Onkologie. Indikation, Technik und Konsequenzen für die Therapieplanung. Urban and Schwarzenberg 1988 with permission from ELSEVIER GmbH, Urban and Fischer Verlag) [12]. (d) Inspection of the situs. Due to Trendelenburg positioning of the patient, the common iliac arteries and the aortic bifurcation are exposed.

The principle of ontogenetically derived surgical compartmental resection may be defined for each organ system. In the following, we will focus on lymphatic basins and the intercalated nodes of the uterus. As outlined by Höckel et al. [3], the lymphatic downstream compartments of the uterine cervix may be divided in the local system, corresponding to intercalated nodes in the vascular mesometrium (mm) and in the regional system corresponding to the paravisceral (pv) and external iliac (ei) basins as primary and the common iliac (ci) and presacral (ps) basins as secondary basins. Tertiary and quaternary basins of the cervix are located along the aorta and caval vein inframesenterically (im) and supramesenterically/infrarenally (sm) (Figures 1(a)–1(c)).

Whereas in cervical cancer skip metastases in tertiary and quaternary basins are extremely uncommon, they occur in endometrial cancer due to the different lymph drainage (Figure 1(c)). Whereas cervical lymph drainage runs downstream along the vascular and fibrofatty mesometria, drainage of uterine corpus involves vascular mesometrium and ovarian vessels. This implicates that in endometrial cancer tertiary and quaternary basins have also to be regarded as primary basins in contrast to cervical cancer.

Following this concept, the following lymph nodes have to be removed in cervical cancer: Lnn. mesometriales, Lnn. Iliaci externi, interni and communes, Lnn. obturatorii, gluteales superiores et inferiores, pudendales, rectales, praesacrales and subaortici corresponding to the primary and secondary basin. In case of proven node metastases in the primary or secondary basin, the inframesenteric periaortic and pericaval nodes ought to be removed; in case of their involvement, the infrarenal periaortic and pericaval nodes in addition.

In case of high risk endometrial cancer without cervical involvement no metastases may be present in the deep gluteal, pudendal, and rectal nodes. However, all other nodes of level 1–4 have to be removed due to the risk of involvement.

The following steps will illustrate the surgical anatomy and operative technique of robotically assisted therapeutic pelvic and periaortic lymphadenectomy (rtLNE).

### 2.5. Therapeutic Pelvic Lymphadenectomy

First, the robotically assisted pelvic therapeutic lymphadenectomy will be described.

**Figure 2:**
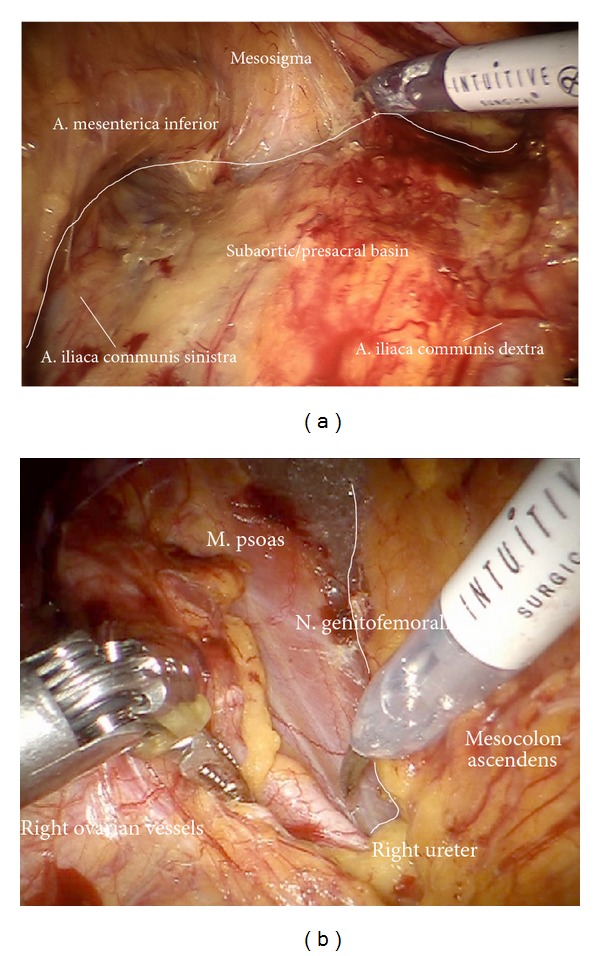
(a) After incision of the parietal peritoneum, the common iliac vessels are exposed. The sigma and mesostigma can be elevated in order to prepare the left iliac vessels further distally. (b) Preparation of the right iliac retroperitoneal space.

*Step *1. **Inspection of the anatomy, exposition of preaortic/precaval region, and incision of peritoneum lateral to the right common iliac artery (Figure 1(d)). Development of the retroperitoneum from the inframesenterial periaortic region to the branching of internal and external iliac vessels and presacrally down to S2. The left common iliac artery can be followed dorsally of the sigmoid mesentery into the “sigmoid tunnel” (Figure 2(a)).

**Figure 3:**
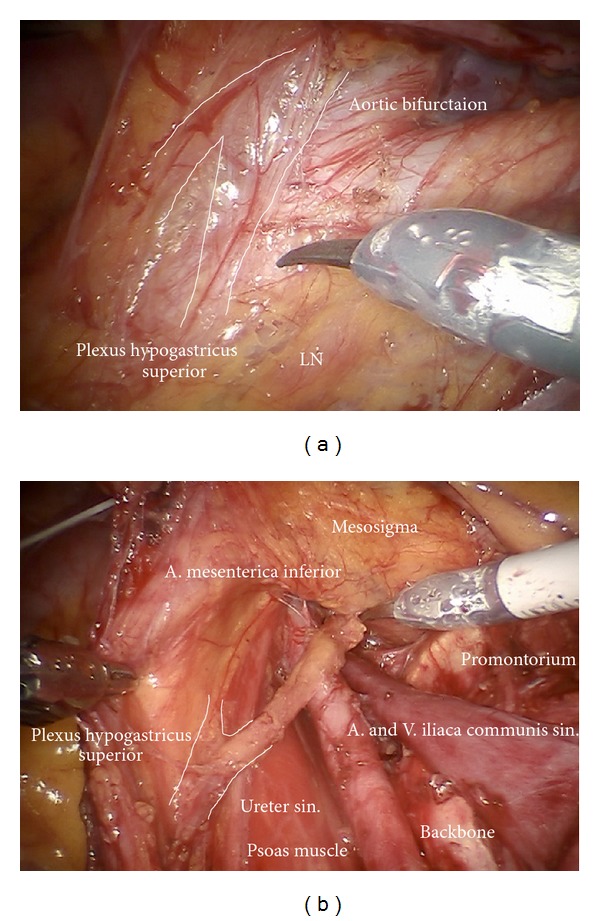
(a) Identification of the hypogastric superior plexus at the aortic bifurcation. (b) Preparation of the hypogastric superior plexus, separating its fibers from vessels and lymphatic basins (ci).

*Step *2. **Separation of the retroperitoneal lymph basins from the mesenteries of the rectosigmoid (Figure 2(a)), ascendant colon (Figure 2(b)), and the hypogastric superior plexus (Figures 3(a) and 3(b)) should define the ventral and lateral borders of common iliac and subaortic/presacral lymph basins.

**Figure 4:**
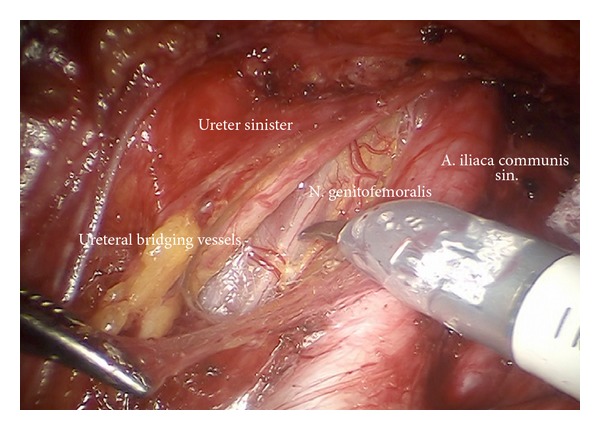
Preparation of the left upper iliac retroperitoneum (ci).

**Figure 5:**
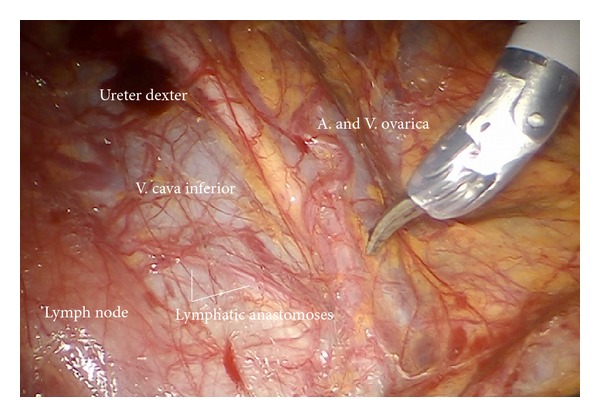
Preparation of the right upper iliac retroperitoneum.

*Step *3. **Preparation of the infundibulopelvic ligaments, ureters, genitofemoral nerves, and psoas muscles on both sides to define the dorsal borders laterally as shown on the left (Figure 4) and on the right (Figure 5).

*Step *4. **Mobilization of the lymphatic tissue “en bloc” starting from laterally, right to medially dissecting vessel anastomoses to the infundibulopelvic ligament and the vena cava, less frequently the aorta; cranially starting from the level of aortic branching, caudally to the iliac branching, usually located at the level of crossing of the ureter. Laterodorsally mobilization of obturator nerve and lumbosacral trunk removing all lymphatic tissue.

**Figure 6:**
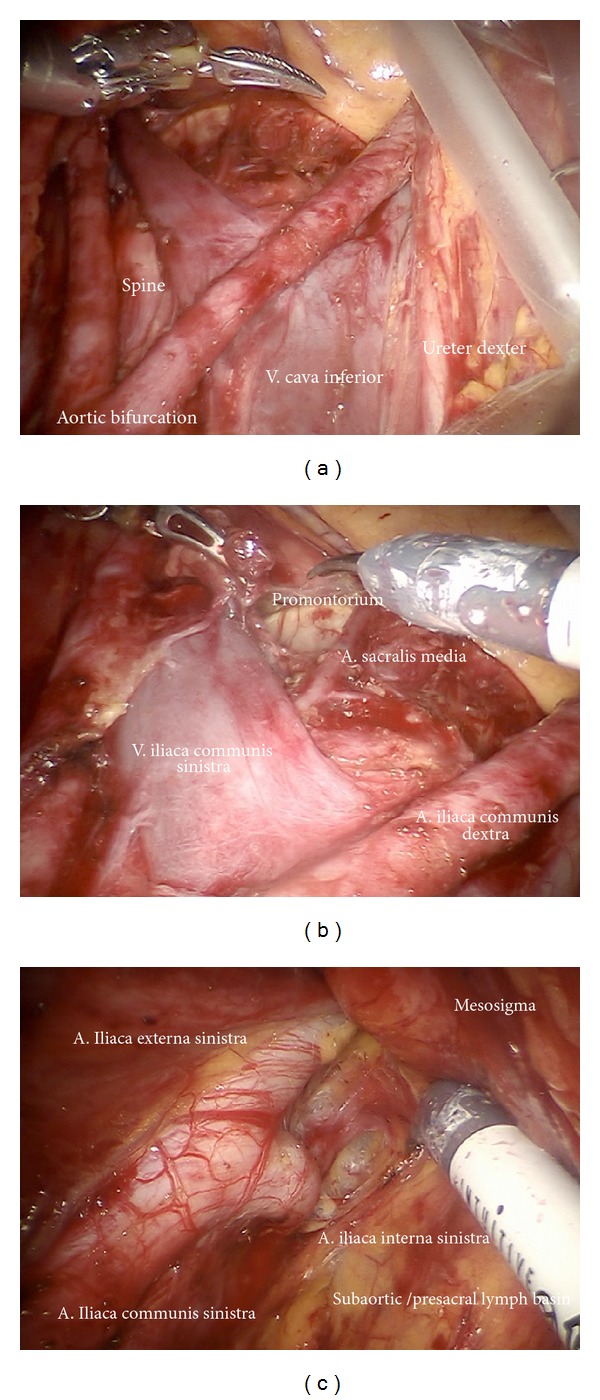
(a) Lymphadenectomy of the upper iliac region exposing the aortic bifurcation (ci, ps). (b) Exposition of the promontorium and the presacral region (subaortic nodes, ps). (c) Exposition and preparation of the left iliac bifurcation.

**Figure 7:**
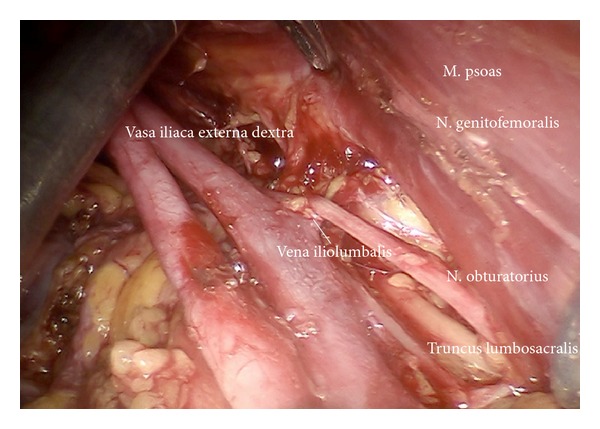
Preparation of the lateral part of upper paravisceral basin (pv) and lower common iliac basin (ci), dissecting all lymphatic tissue.

*Step *5. **Dissection of the lymphatic basin and separation from the medial border of the common iliac vessels down to the branching of the internal iliac artery into its anterior and posterior branches, medially down to S2. Mobilization from caudally to cranially by exposing the promontory, the median sacral artery, and the back bone (Figures 6(a)–6(c)). Laterally and dorsally of the common iliac vessels, mobilization of obturator nerve, and lumbosacral trunk removing all lymphatic tissue (Figure 7).

*Step *6. **Same preparation on the left side from medially to laterally. The preparation of the branches of the internal vessels may be performed down to the left uterine artery due to the excellent access via the “sigmoid tunnel.” Now the mobilized tissue of the secondary compartment (level II) may be resected and preserved in an endobag or left “en bloc.”

**Figure 8:**
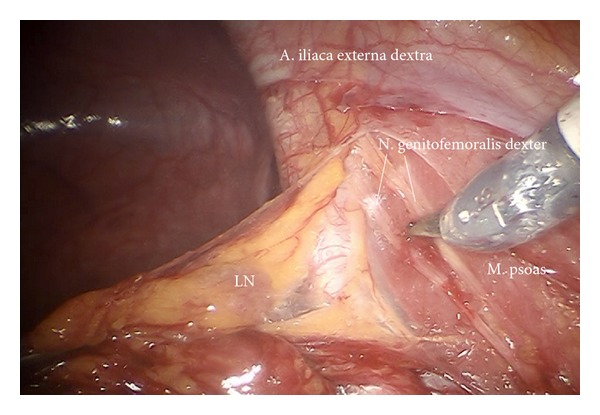
Preparation of the right external iliac vessels (ei).

**Figure 9:**
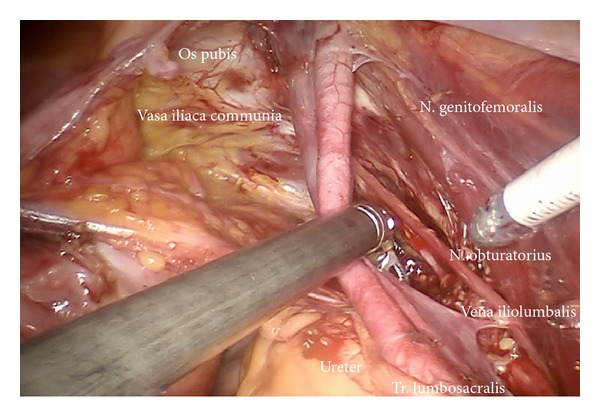
Preparation of lateral part of paravisceral basin (exposition of obturator nerve).

**Figure 10:**
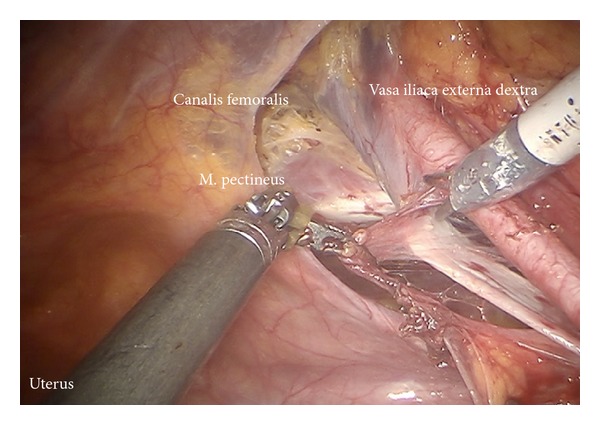
Lymphadenectomy in between right external iliac artery and vein (ei).

**Figure 11:**
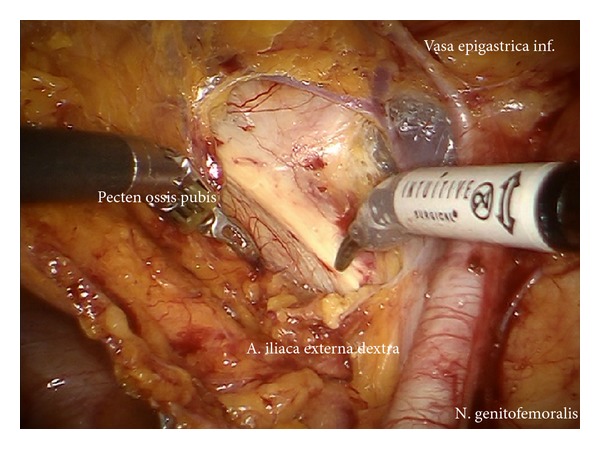
Caudal pelvic preparation on the right side exposing the pubic bone (ei).

*Step *7. **Exposition of the right external iliac region and again preparing the genitofemoral nerve and the psoas muscle laterally (Figure 8) down to the A. and V. circumflexa ilium profunda and the level of the femoral channel. Mobilization of lymph tissue from laterally to medially by exposing the external iliac vessels. Opening the paravisceral basin from lateral by dissecting the nodal tissue from the obturator fascia down to the arcus tendineus pushing the iliac vessels medially. Dissecting the lymphatic tissue at the level of the femoral channel preserving the Vasa circumflexa ilium profunda and the inferior epigastric vessels medially (ei) (Figures 9, 10, and 11). 

**Figure 12:**
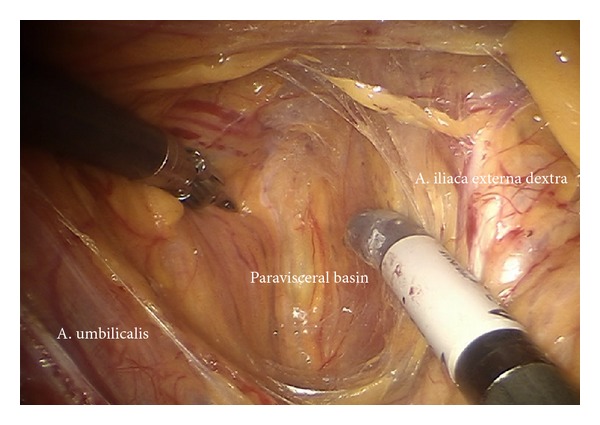
Development of the paravisceral space by medialization of the vesical mesenteric septum and dissection down to the endopelvic fascia (pv).

**Figure 13:**
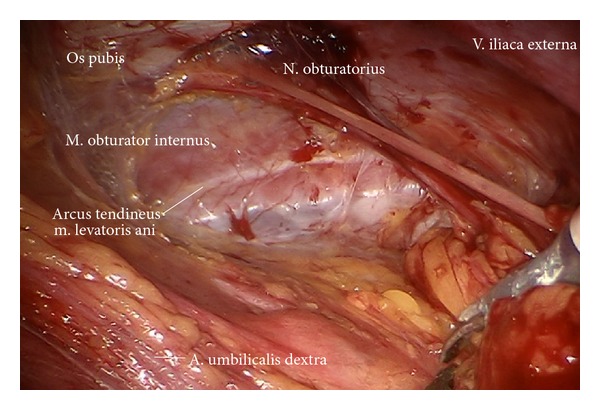
Clearing of the lateral and medial part of the paravisceral basin down to the pubococcygeal and iliococcygeal muscles and up to the internal iliac vessels (pv).

*Step *8. **Identification of the umbilical artery (medial umbilical ligament) and preparation from the anterior abdominal wall to its origin from the internal iliac artery. Development of the paravisceral space from medially by medialization of the vesical mesenteric septum and dissection down to the endopelvic fascia (Figure 12). Removal of the medial nodes located in front of the femoral channel and exposition of the pecten ossis pubis (Figure 11). By preparing down along the obturator muscle the obturator nerve/vessel bundle can be identified, freed completely from lymph tissue and preserved. Now the lateral and medial part of paravisceral basin can be joined and totally be cleaned down to the pubococcygeal and iliococcygeal muscles and up to the internal iliac vessels (pv, caudally) (Figure 13).

**Figure 14:**
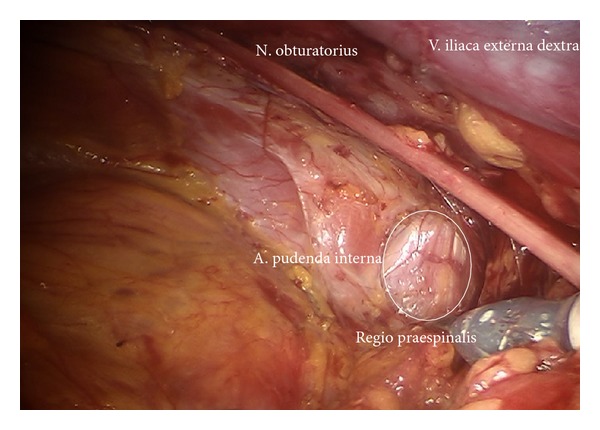
Preparation of the right prespinal region with corresponding lymph basins (pv).

**Figure 15:**
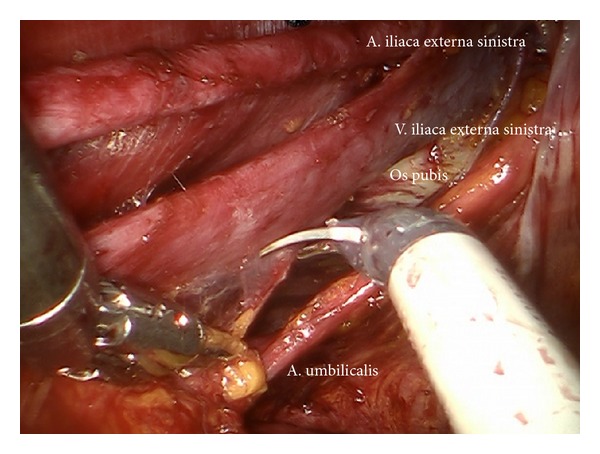
Deep pelvic preparation on the left side (ei, pv).

**Figure 16:**
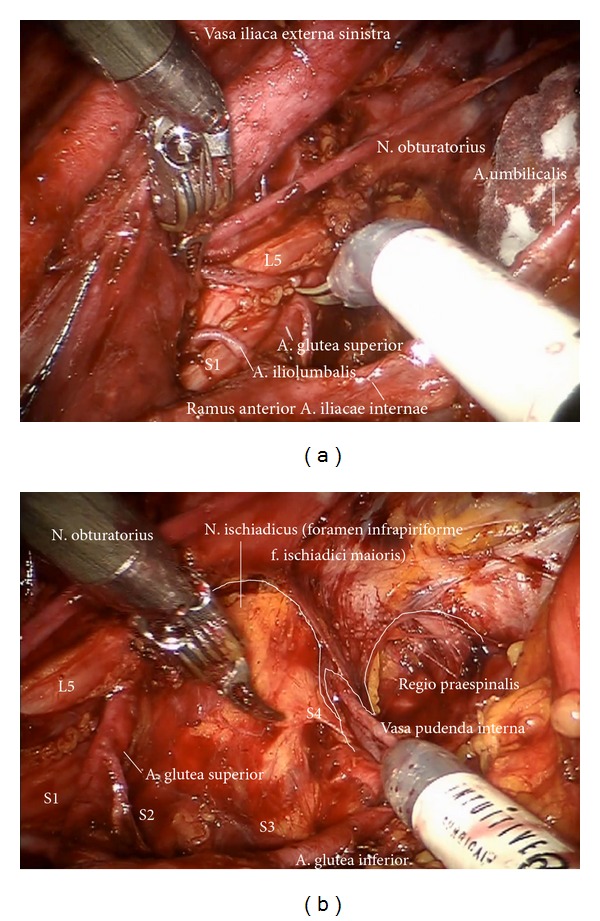
(a) Exposition of the superior gluteal artery and lumbosacral nerve roots (pv). (b) Visualization of the N. ischiadicus and the Vasa pudenda (upper paravisceral basin).

*Step *9. **In case of removal of the lymphatic tissue along the branches of iliac vessels in cervical cancer first the prespinal tissue will be removed exposing the pudendal artery reentering the pelvis via the minor ischiadic foramen (Figure 14). Step by step first the arterial branches of the Aa. glutea superior, inferior, iliolumbalis, rectalis media and pudenda interna, and the corresponding veins will be freed of lymphatic tissue, completely. Concomitantly the lumbosacral trunc, the roots of S1–S4 of sacral plexus, the ischiadic, and pudendal nerve are presented and preserved ((Figures 15, 16(a), and 16(b)) shown on the left) (pv, cranially).

Steps 10–12 correspond to steps 7–9 on the left side.

**Figure 17:**
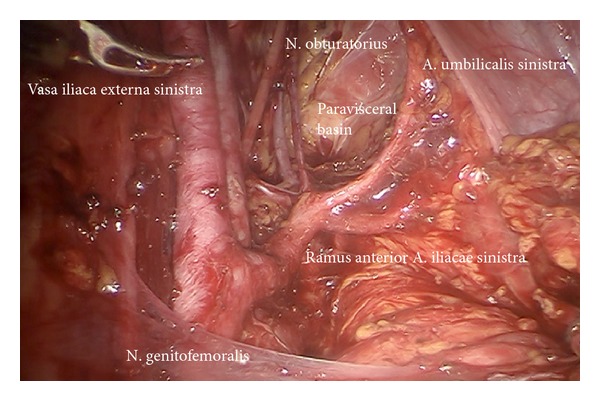
Left lower paravisceral basin (pv).

**Figure 18:**
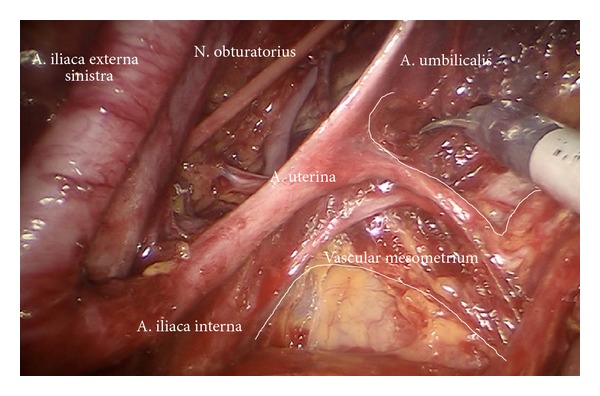
Left vascular mesometrium (mm) with exposed uterine vessels.

**Figure 19:**
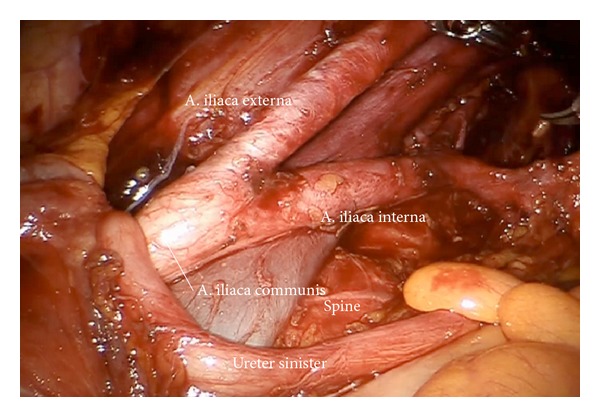
Left iliac bifurcation, border to common iliac basin (ci, line).

Following preparation of the branches of the anterior internal iliac artery (Figure 17) the mesometrial nodes are usually removed concomitantly with resection of vascular mesometrium by TMMR in cervical or PMMR in endometrial cancer (Figure 18). Finally, the connection to the left common iliac region has to be established to guarantee complete node dissection also posterior to the sigmoid colon (Figure 19). Thus, the therapeutic pelvic lymphadenectomy is completed.

### 2.6. Periaortic Lymphadenectomy

In case of indication for periaortic lymphadenectomy two different situations have to be addressed: primary tumor involves uterine corpus and/or adnexa or the cervix only. 

In the first situation lymph drainage follows the vascular mesometrium and the infundibulopelvic ligament; therefore primary lymph basins are pelvic and periaortic, simultaneously, and therefore have to be removed entirely. In the second situation periaortic lymph basins are of third or forth order and may be maintained in case of histologically proven negative pelvic nodes.

In case of tumor involvement of the uterine corpus or the adnexa in addition to the vascular mesometrium also the network for vascular and lymphatic anastomoses between uterus and adnexa has to be removed, best covered by the peritoneal leaves of the broad ligament (PMMR). The vascular and lymphatic drainage system along the ovarian vessels has to be removed in analogy to the vascular mesometrium up to its connection to the periaortic and pericaval lymph basins. Whereas the vascular anastomoses to the mesenteric vessel system (ascendant and descendant colon) are dissected the connections to the periaortic lymph basins are preserved and removed together “en bloc.” 

On the other hand, if tumor is located in uterine corpus, exclusively, the deep gluteal, pudendal, and rectal nodes may be preserved, since usually there will be no “upstream drainage” in such situation except for clinically suspicious nodes. On the other hand, if it is located in uterine cervix only, periaortic lymphadenectomy has only to be performed in case of positive pelvic nodes, and resection of the ovarian vessel system is not mandatory.

**Figure 20:**
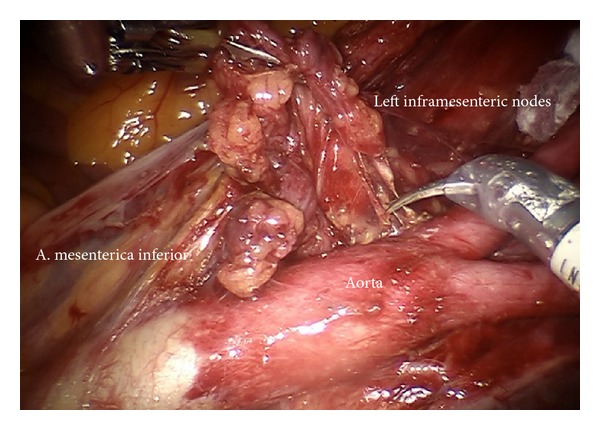
Preparation of the left inframesenteric lymph nodes (im).

**Figure 21:**
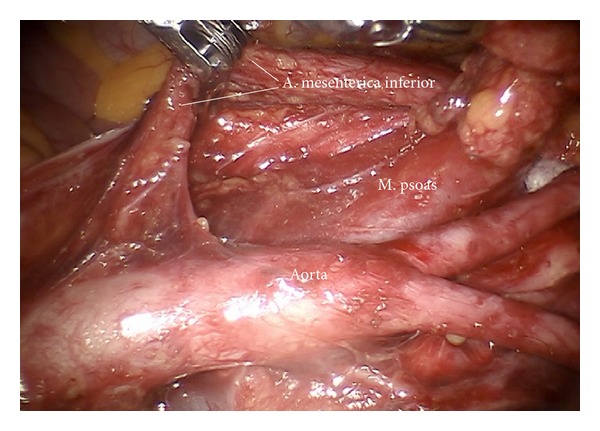
Preparation of the lower periaortic region exposing the inferior mesenteric artery (im).

**Figure 22:**
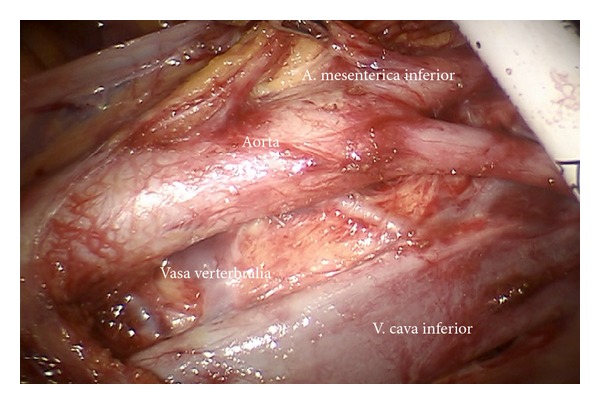
Interaortocaval region with vertebral vessels (ir and im).

**Figure 23:**
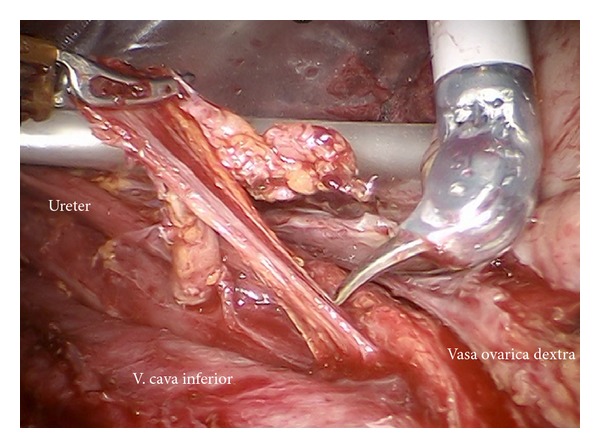
Upper periaortic region with right ovarian vessels (ir).

*Step 13.* In case of cervical cancer inframesenteric periaortic lymphadenectomy may be performed following elevation of the superior hypogastric plexus, the inferior mesenteric artery, and the sigmoid colon ventrally, thus continuing lymphadenectomy from common iliac vessels (Figures 20 and 21). It has to be ascertained to remove the interaortocaval nodes and the chains dorsally of vena cava and aorta (Figures 22 and 23). 

**Figure 24:**
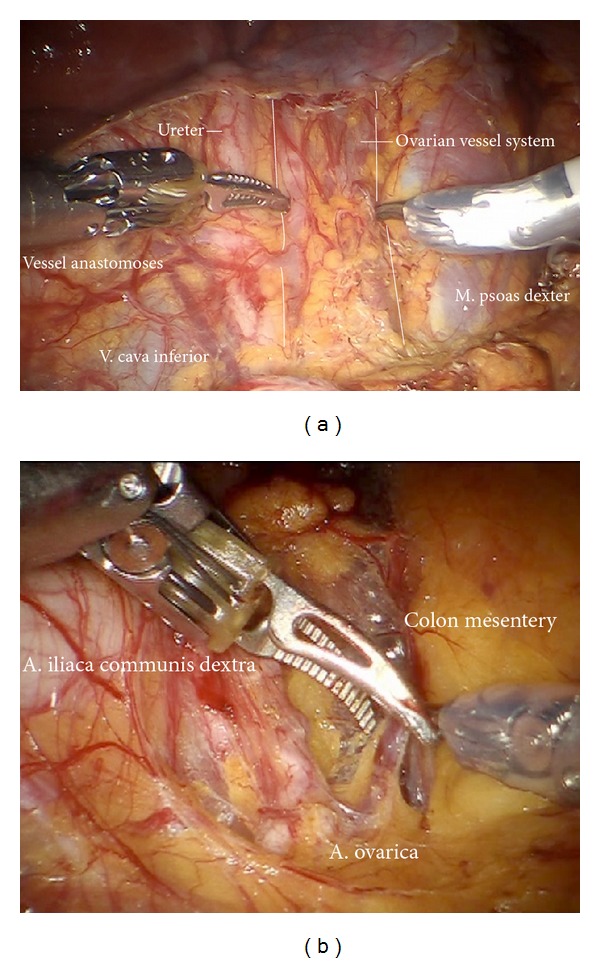
(a) Preparation of the ovarian vessels next to the ureter. (b) Separation of the left ovarian vessel system from the mesostigma.

**Figure 25:**
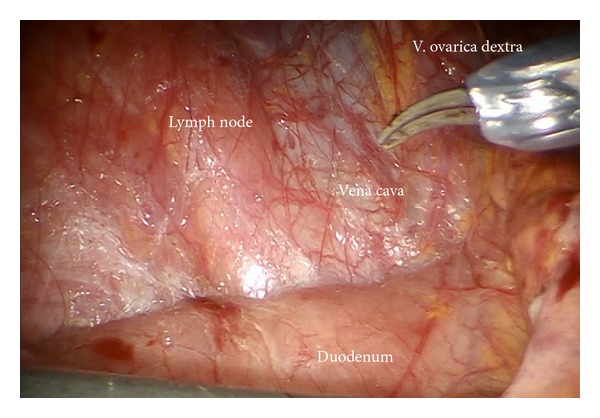
Upper periaortic region, mobilization of the duodenum.

*Step 14.* In case of endometrial or ovarian cancer in any case the infundibulopelvic ligaments have to be resected first; aorta, vena cava, the right ureter, and the right infundibulopelvic ligament (Figure 24(a)) are identified. Mobilization of the right colic mesentery allows to identify vessel anastomoses to the ovarian vessel system and can be divided (Figure 24(b)). Following the ovarian vein the lateral border of the ovarian lymphovascular drainage bundle can be followed until the right angle between vena cava and right renal vein is reached. Thus all anastomoses to the periaortic lymph basin will be kept intact. The duodenum usually is loosely attached to the preaortic region and has to be mobilized (Figure 25).

**Figure 26:**
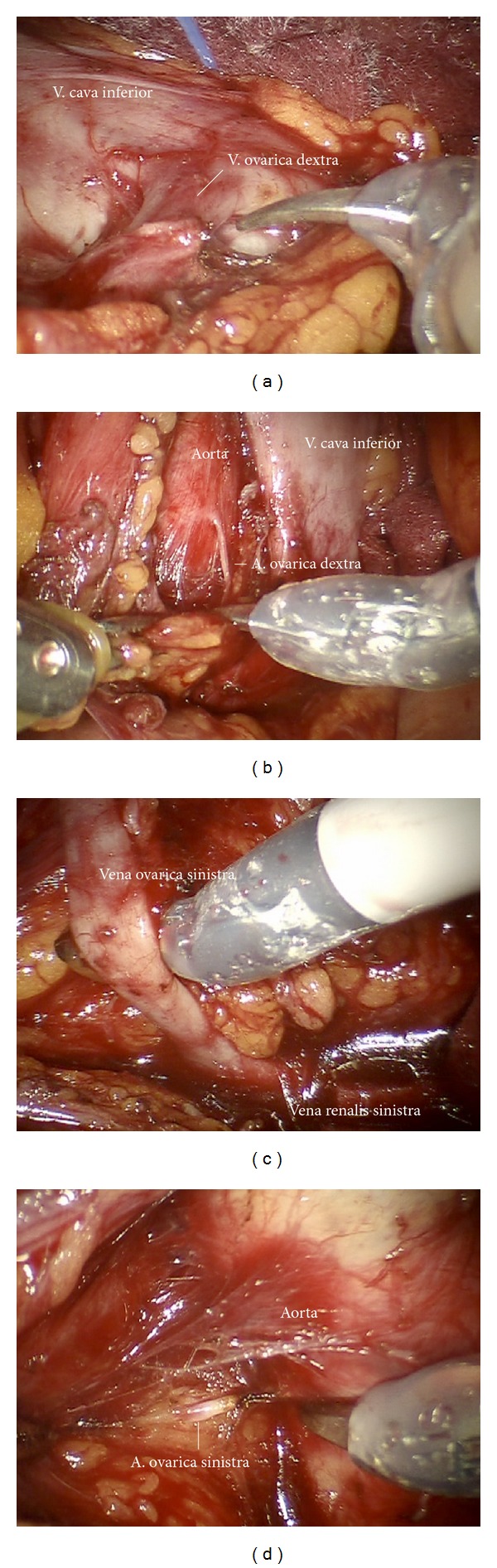
(a) Preparation and dissection of the right ovarian vein (ir). (b) Exposition of the right ovarian artery at its origin (ir). (c) Preparation of the left ovarian vein (ir). (d) Exposition of the left ovarian artery (ir).

*Step 15.* The right ovarian vein is isolated at the level of caval vein and dissected (Figure 26(a)). Now the lymphatic channels may be dissected cranially along the right renal vein and dorsally to the right paracaval region to expose the back bone. The lymphatic paraaortic system may now be pulled to the left, and the interaortocaval region will be exposed. The vertebral vessels will be freed from lymphatic tissue, and the vena cava now is cleaned at 360 degrees. Care has to be taken to remove the dorsolateral lymph node chain running draining from deep right pelvis, which is not directly connected ventral chain. The right ovarian artery will be exposed, coagulated and divided (Figure 26(b)). The left renal vein will now be identified at its caval angle and followed to lateral, and again the lymph channels will be dissected along the left renal vein until the left ovarian vein can be identified. The left ovarian vein is coagulated, and cut (Figure 26(c)). Now moving downward using the ovarian vein as lateral border and the aorta as medial border for mobilization and resection of left periaortic lymphatic basin left ovarian artery will be identified approximately at the level of right ovarian artery, coagulated, and cut (Figure 26(d)). 

**Figure 27:**
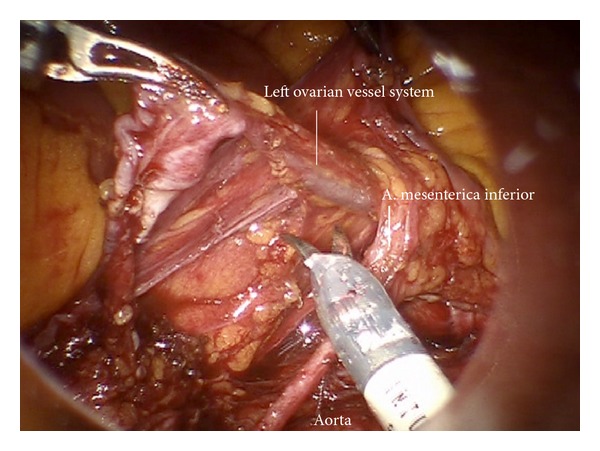
Separation of the left periaortic lymphatic tissue together with the left infundibulopelvic ligament from the inferior mesenteric system (im).

**Figure 28:**
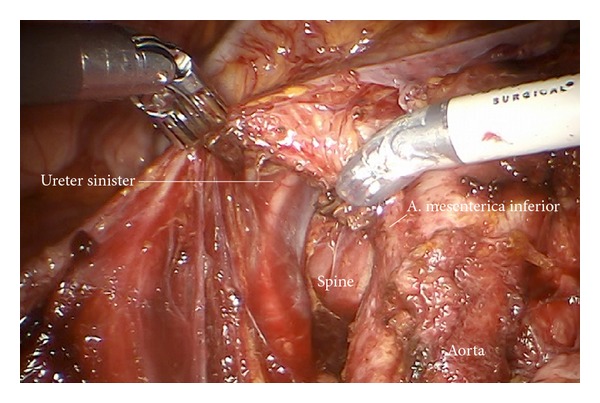
Preparation of the left periaortic lymphatic tissue with special attention to fibers of the superior hypogastric plexus.

**Figure 29:**
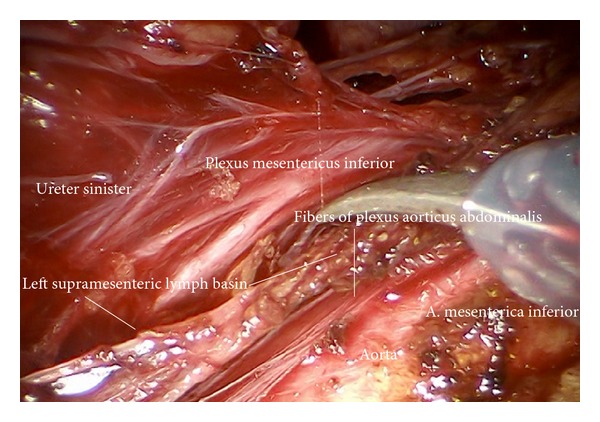
Fibers of the inferior mesenteric and superior hypogastric plexus are clearly visible; they are mobilized together with the mesostigma (ir).

*Step 16.* The left periaortic lymphatic tissue together with the left infundibulopelvic ligament can be separated from the inferior mesenteric system (Figure 27) and the superior hypogastric plexus. To do this the mesenteric vessels are elevated ventrally together with the nerve bundles, and the lymphatic tissue is mobilized and pushed through the paraaortic “tunnel” posterior to the mesenteric artery caudally (Figure 28). Doing this, attention has to be paid to preserve the nerve fibers of the inferior mesenteric plexus and the aortic plexus which can be separated from the lymphatic tissue (Figure 29). The right part, especially the right sided crossing nerve fibers of the aortic plexus, however, for our experience cannot entirely be preserved in case of therapeutic periaortic lymphadenectomy.

**Figure 30:**
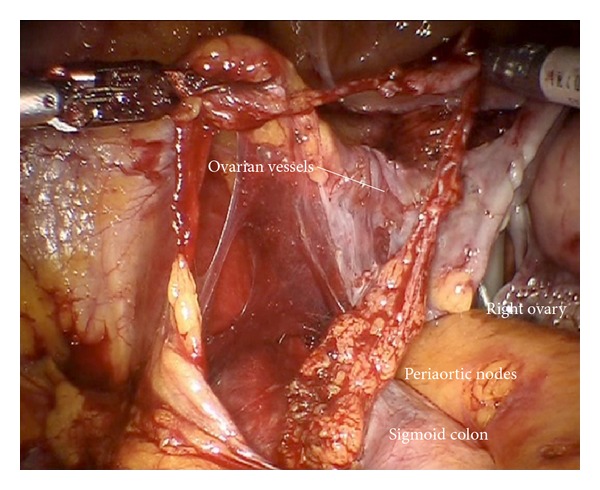
En bloc mobilization of the left infundibulopelvic ligament through the “sigmoid tunnel.”

*Step 17.* The left infundibulopelvic ligament and periaortic lymph tissue are mobilized inframesenterically using the left aortic wall, the back bone, the sympathetic chain, and the left ureter as border, medially, dorsally, and laterally. It may be developed en bloc from caudally through the “sigmoid channel” (Figure 30).

If resection of the ovarian vessel drainage system is not necessary—in case of cervical cancer—the procedure can be performed identically following dissection of connecting vessels to the periaortic drainage system and lateralization of the ovarian vessels.

During the preparation care should be taken to mobilize the entire lymphatic basins of interest and concomitantly preserve the autonomic nerve fibers and the ureteral supplying vessels as thoroughly as possible.

This compartment defined lymphadenectomy in therapeutic intention is combined by compartment defined local surgery, for example, TMMR (total mesometrial resection) or FMMR (fertility-preserving mesometrial resection) for cervical cancer, PMMR (peritoneal mesometrial resection) for endometrial cancer or salpingo-oophorectomy with resection of ovarian vascular/lymphatic vessel system in early ovarian cancer. It is aimed to facilitate locoregional tumor control without additional radiotherapy.

## 3. Results

In total, 35 patients were operated with the diagnosis of endometrial (*n* = 16) or cervical (*n* = 19) cancer. All patients received a total mesometrial (cervical cancer) or a peritoneal mesometrial (endometrial cancer) resection of the uterus and therapeutic pelvic lymphadenectomy (cervical cancer) or therapeutic pelvic and periaortic lymphadenectomy (endometrial cancer).

The majority of endometrial as well as cervical cancer patients were diagnosed with FIGO I.

No transition to open surgery was necessary due to complications or technical problems. We noted complication rates of 13% for endometrial cancer and 21% for cervical cancer. In the group of endometrial cancer patients, one patient had a superficial vein thrombosis, and one had a phase of transitional postoperative aphasia which resolved completely. In the group of cervical cancer patients, there were two reoperations due to formation of an intra-abdominal lymphocele/abscess and one due to postoperative bleeding. One patient had a port infection which was treated by local aseptic measures.

Endometrial cancer patients with tumor stage FIGO III and/or positive lymph nodes were treated with six cycles of adjuvant platinum based chemotherapy. Patients with cervical cancer and positive lymph nodes received an adjuvant platinum based chemotherapy with or without irradiation.

Median followup was 20(22) month (Table 2), and up to now 1 patient with cervical cancer and 2 patients with endometrial cancer developed recurrent disease. The patient with recurrence of cervical cancer presented with stage of pT2b, pN1, and G2 and had rupture of the anterior cervical fascia during surgery with exposure of tumor to the surgical field. She refused radiation therapy and developed trocar site recurrence after 9 months, treated by radiation therapy and surgical excision, respectively. The first patient with endometrial cancer recurrence was a 26-year-old woman with massive overweight (BMI 57 kg/m^2^). The initial tumor stage was pT1a, N0 (0/29), and G3, and the histopathology revealed an endometrioid tumor type. She did not receive adjuvant treatment and presented one year after initial diagnosis with liver metastases; there was no evidence of relapse in the pelvis. The second patient with recurrence of endometrial cancer had initially a tumor stage of pT3b, pN1 (24/46), and G2 (endometrioid histopathology). The tumor was completely resected, and the patient received an adjuvant chemotherapy with carboplatinum AUC 5 and paclitaxel 175 mg/m^2^ d1, q3w over six cycles. After 11 months, she presented with local pelvic recurrence and liver metastases and was treated with palliative chemotherapy.

No patient, up to now, neither in cervical nor in endometrial cancer developed isolated locoregional recurrence.

Detailed data on patient characteristics and oncological outcome are depicted in Tables 1 and 2.

## 4. Discussion

We were able to show that the principle of compartment based surgery in uterine cancers, which is applied in the techniques of modified radical hysterectomies as TMMR and PMMR, can be translated with respect to the removal of the accordant lymphatic basins, to minimal invasive, robotically assisted procedures.

The method appears to be feasible and safe, especially if we consider that the presented data were collected during the very first learning curve of the authors. Although the followup time of our patients is limited, we are confident that the oncological outcome will be comparable to open surgery. Due to the excellent visibility, the high precision, and control of preparation, the technical radicalness of lymph node dissection can be controlled individually and documented continuously for each single region allowing complete removal of tissue at least comparable to open surgery if not even better. 

In our experience, patients grossly benefit from these procedures. First, compartment based surgery oncologically appears to exert excellent locoregional control combined with a lower complication rate especially regarding the autonomous nerve plexus. Second, the translation to a minimal invasive method by robot assistance adds the advantages of minimal invasive surgery, regarding blood loss, mobilization, length of hospitalization, and short-term complications [14, 15]. However, exposure of tumor to the surgical field has strictly to be avoided, as it is in classical laparoscopic surgery.

In conclusion, we suggest that the minimal invasive approach of compartment based oncologic surgery for uterine cancers by robotic assistance is feasible, safe, and beneficial for patients. To evaluate whether the excellent monocentric data with excellent locoregional tumor control and low morbidity of Höckel in cervical cancer [3, 9, 10] holds true in a multicenter approach independent of open or laparoscopic access, a multicenter observational study has been initiated, which will start recruitment in 2013.

However, for surgical studies it is crucial to define exactly the surgical procedure to be done. For “therapeutic lymphadenectomy” the technique described in this publication will be the reference basis, as it could be for other trials referring to “therapeutic lymphadenectomy.” In addition, operative procedure of TMMR and tLNE is exactly defined by surgical videos of open and robotically assisted TMMR and therapeutic lymphadenectomy preceded by education at Leipzig School of Radical Pelvic Surgery and followed by onsite visit to control for the technique. In future, from our point of view, this kind of preparation and auditing of surgical studies could contribute to comparability between different sites, which is not only mandatory in scientific studies but may also beneficial in education and clinical practice. 3 D Vision, HD Quality, magnification of structures, and thus, excellent quality of surgical videos derived from robotic surgery will revolutionize our prospects for education, scientific research, and quality control.

## Figures and Tables

**Table 1:** Detailed information on clinical and perioperative data.

	Patients	Age (years)	BMI (kg/m^2^)	Number of lymph nodes removed	Hospital stay (days)	Blood transfusion	Complication rate
Endometrial cancer	16	59 (26–78)	32 (20–57)	32 (16–52)	10 (5–25)	1 6%	2 13%
Cervical cancer	19	46 (31–71)	23 (18–33)	34 (13–68)	9 (6–33)	2 11%	4 21%

**Table 2:** Detailed information on oncological data.

	Patients	Tumor stage			Grading			Positive lymph nodes	Recurrent disease	Median Follow-up (months)
		FIGO I	FIGO II	FIGO III	G1	G2	G3
Endometrial cancer	16	13 (81%)	0	3 (19%)	0	12 (75%)	4 (25%)	4 (25%)	2 (13%)	22 (13–28)
Cervical cancer	19	18 (95%)	1 (5%)	0	2 (11%)	14 (74%)	3 (16%)	5 (26%)	1 (5%)	20 (12–28)
